# The brain-gut axis in autism spectrum disorder: from gastrointestinal dysfunction to neuroimmune mechanisms

**DOI:** 10.3389/fpsyt.2026.1907757

**Published:** 2026-08-13

**Authors:** Fei Fan, Bo Wang, Fei Han

**Affiliations:** Department of Paediatrics, Guang’anmen Hospital, China Academy of Chinese Medical Sciences, Beijing, China

**Keywords:** autism spectrum disorder, gastrointestinal dysfunction, gut-brain axis, microbiota, neuroinflammation, probiotics

## Abstract

Autism spectrum disorder (ASD) is a neurodevelopmental condition characterized by social deficits and repetitive behaviors, often accompanied by gastrointestinal (GI) symptoms such as constipation, diarrhea, and abdominal pain. Emerging evidence highlights the gut-brain axis as a key pathway linking GI dysfunction to ASD pathophysiology. This review synthesizes current findings on the interplay among dietary patterns, gut microbiota dysbiosis, intestinal barrier dysfunction, and neuroimmune activation in ASD. We also discuss shared genetic vulnerabilities and environmental factors influencing both gut and brain function. Finally, we evaluate microbiota-targeted interventions, including probiotics and fecal microbiota transplantation, as potential therapeutic strategies. Understanding these mechanisms may inform personalized approaches to managing GI and behavioral symptoms in ASD.

## Introduction

ASD is a complex neurodevelopmental condition characterized by social communication deficits and repetitive behaviors. It affects approximately 1 in 31 children in the United States, with an increasing global prevalence (1). Beyond the core diagnostic features, individuals with ASD frequently experience comorbidities, with gastrointestinal (GI) symptoms being among the most common, affecting approximately 48. 7% of affected individuals, including constipation (26. 2%), diarrhea (19. 9%), abdominal pain (21. 4%), and food selectivity (59. 1%) (2). These symptoms persist over time and correlate with increased ASD severity and behavioral challenges. Children with ASD and GI symptoms exhibit more irritability, social withdrawal, and anxiety than those without GI symptoms, suggesting that abdominal discomfort may exacerbate behavioral manifestations (3).

The gut-brain axis, a bidirectional communication system involving neural, endocrine, immune, and metabolic pathways, has emerged as a critical framework for understanding the connection between GI dysfunction and ASD symptomatology (4). The gut-brain axis encompasses multiple communication pathways, including the vagus nerve, enteric nervous system, hypothalamic-pituitary-adrenal (HPA) axis, and immune signaling mediated by microbial metabolites and cytokines (5). Disruptions in these pathways have been increasingly implicated in the pathophysiology of ASD (6). Moreover, microbial metabolites such as short-chain fatty acids (SCFAs), neurotransmitters, and immunomodulatory molecules produced in the gut can influence brain function and neurodevelopment (7). These findings suggest that GI dysfunction in ASD is not merely a peripheral comorbidity but may actively contribute to the core and associated symptoms of the disorder.

In this review, we synthesize current evidence on the role of the gut-brain axis in ASD, with a focus on GI dysfunction, microbiota composition, and neuroimmune mechanisms. We propose an integrative framework that positions these components within a self-perpetuating cycle and offer a critical evaluation of recent therapeutic strategies, including RCTs of fecal microbiota transplantation (FMT).

## Search strategy

For this review, we searched PubMed and Web of Science for articles published up to January 2026. The search terms included combinations of the following keywords: “autism spectrum disorder,” “gut-brain axis,” “gastrointestinal dysfunction,” “microbiota,” “intestinal permeability,” “probiotics,” and “dietary interventions.” We included peer-reviewed original research, systematic reviews, and meta-analyses published in English.

## Gastrointestinal dysfunction in ASD

GI dysfunction is a significant and prevalent comorbidity in ASD, profoundly affecting the quality of life of affected individuals (8). The etiology of GI symptoms is multifactorial and involves a complex interplay between dietary patterns, gut microbiota, and neuroimmune function (3, 9). Food selectivity is a primary behavioral contributor to GI issues in ASD. It is often driven by sensory aversion to specific textures, tastes, or smells (9, 10). This problematic eating behavior affects a substantial proportion of children with autism, with reported rates as high as 60. 6% (10, 11). Food selectivity can lead to a diet low in fiber and rich in processed, energy-dense foods, which directly contribute to constipation and other functional GI disorders (12, 13). This restricted intake may also cause micronutrient deficiencies, such as vitamins A, D, and B complex deficiencies, further compromising gut health and immune regulation (14–16).

Beyond dietary intake, intrinsic biological factors play a critical role in the GI pathology observed in ASD (17). The underlying gut pathology, independent of diet, has been consistently documented (18). Histological examinations of GI-symptomatic children with ASD frequently reveal a unique nonspecific ileocolitis (19). This condition is characterized by lymphocytic infiltration in both the small and large intestines, which is distinct from classical inflammatory bowel disease (20). This low-grade inflammation is often accompanied by increased intestinal permeability, commonly referred to as “leaky gut” (21, 22). A “leaky gut” allows bacterial products and dietary antigens to cross the intestinal barrier and enter systemic circulation (23). This translocation of substances can activate neuroimmune pathways, leading to systemic inflammation (24, 25). The resulting low-grade systemic inflammation can, in turn, exacerbate both GI symptoms and behavioral challenges, creating a self-perpetuating cycle (26, 27).

## The gut microbiota in ASD pathophysiology

The dysbiosis observed in ASD is often marked by a reduced Bacteroidetes-to-Firmicutes ratio and overgrowth of potentially pathogenic bacteria such as *Clostridium* and *Desulfovibrio* species, along with a notable depletion of beneficial genera such as Bifidobacterium and Prevotella (28–30). These microbial alterations are not merely associative but can directly affect host physiology. For instance, increased *Clostridium* abundance contributes to elevated levels of propionic acid and the neurotoxin p-cresol, both linked to ASD symptom severity, while an overabundance of *Desulfovibrio* can produce cytotoxic hydrogen sulfide, potentially exacerbating gastrointestinal inflammation (31). Conversely, a reduction in *Bifidobacterium* compromises gut barrier integrity and reduces the synthesis of neurotransmitters such as GABA (32, 33). The fungus *Candida albicans* has also been found in higher abundance in individuals with ASD, where it may produce behavior-influencing toxins, such as ammonia (34).

Mechanistically, these microbial differences contribute to GI dysfunction and neurological symptoms through several pathways. Germ-free mice colonized with microbiota from ASD donors develop ASD-like behaviors, demonstrating that the microbiota is sufficient to induce these phenotypes in animal models (35). However, dietary patterns, particularly food selectivity, may independently shape both gut microbial community structure and behavioral phenotypes (9, 10). This independent influence positions diet as a potential confounder in the observed associations, complicating causal inference. Microbial metabolites play a central role. For example, short-chain fatty acids such as propionate, which are often overproduced by *Clostridia* species, can induce autism-like behaviors in animal models through the promotion of neuroinflammation and the disruption of neurotransmitter signaling pathways. This effect stands in contrast to the general anti-inflammatory actions of butyrate, which support intestinal barrier integrity and exert protective functions within the gut–brain axis (35, 36). Additionally, components of gram-negative bacteria, such as lipopolysaccharide (LPS), can translocate across the “leaky gut” into systemic circulation (37). LPS activates toll-like receptor 4 (TLR4), triggering the release of pro-inflammatory cytokines that can cross the blood-brain barrier and contribute to neuroinflammation (38–40). Collectively, these findings indicate that the gut microbiota influences brain function and behavior by modulating neurotransmitter systems and immune responses and producing neuroactive or inflammatory compounds.

## Neuroimmune mechanisms

The gut microbiota regulates intestinal barrier integrity and immune function, and increased intestinal permeability in ASD allows bacterial products, such as lipopolysaccharides, to enter the circulation, triggering systemic inflammation (41). Once in the circulation, LPS, as a pathogen-associated molecular pattern, is recognized by pattern recognition receptors such as TLR4 on immune cells (42). This recognition triggers a cascade of immune responses, leading to the systemic release of pro-inflammatory cytokines, including IL-1β, IL-6, and TNF-α (43). Research has substantiated this link, showing that children with ASD and comorbid GI symptoms exhibit significantly higher levels of circulating pro-inflammatory cytokines than those with ASD alone or typically developing children, providing direct evidence of peripheral immune dysfunction in this subgroup (44).

These peripherally generated cytokines can influence the central nervous system (CNS) through multiple pathways and cross the blood-brain barrier (BBB) via active transport mechanisms or act on endothelial cells in circumventricular organs (areas lacking a complete BBB) to induce the production of secondary messengers (45, 46). Sustained systemic inflammation can also compromise the BBB integrity, thereby increasing its permeability (24). Once within the CNS, these inflammatory signals activate the brain’s resident immune cells, such as microglia and astrocytes. Postmortem studies of individuals with ASD have provided key evidence for this neuroinflammation, consistently revealing activated microglia and astrocytes in multiple brain regions, particularly in the frontal cortex and cerebellum, areas crucial for social behavior, communication, and motor coordination (47, 48). Activated glial cells release a host of inflammatory mediators including cytokines, chemokines, and complement proteins (44, 49). Although these molecules aim to defend against potential threats in the context of ASD, they negatively affect neuronal function by disrupting the fine structure and plasticity of synapses and altering the delicate balance between excitatory and inhibitory neurotransmission (50, 51). This neuroinflammatory cascade is critically regulated by NF-κB signaling, a pathway centrally involved in various psychiatric disorders including ASD (52). Furthermore, Rose et al. have underscored the importance of local, gut-specific immunity (53). They discovered that children with ASD and GI symptoms exhibit a distinct profile of elevated mucosa-relevant cytokines compared to children with ASD without GI symptoms and typically developing controls. This finding suggests that chronic, localized intestinal inflammation is an integral component of ASD pathogenesis, rather than merely a passive consequence of systemic inflammation. This local immune activation likely exacerbates the breakdown of the intestinal barrier, perpetuating a vicious cycle of inflammation that extends from the gut to the brain and potentially back again, as stress and behavior can further influence gut function.

## Shared genetic and environmental risk factors

Many high-confidence ASD genes are expressed in both the brain and GI tissues, including CHD8, SHANK3, and SLC6A4 (serotonin transporter). Mice expressing ASD-associated serotonin transporter variants exhibit behavioral abnormalities and GI dysmotility (54). Environmental factors, including maternal immune activation, antibiotic exposure, and dietary factors, also influence gut microbiota composition and ASD risk. Genetically, ASD and GI disorders share a significant overlap in susceptibility loci, and bioinformatic analyses integrating data from DisGeNET and genome-wide association studies have identified common genetic variants associated with both ASD and inflammatory bowel or celiac diseases. The key genes implicated include those involved in immune regulation and gut barrier integrity (55). The CNTNAP2 gene, for instance, has been robustly linked to ASD risk, and integrative multiomics studies have revealed that its disruption affects the molecular networks governing both neuronal connectivity and gut physiology (56). Similarly, mutations in genes such as CHD8, which are primarily associated with neurodevelopmental phenotypes, also correlate with GI symptoms, suggesting pleiotropic effects across organ systems (57).

This genetic architecture is further complicated by epigenetic modifications that mediate gene-environment interactions. Prenatal and early-life environmental exposures can induce DNA methylation changes in immune- and neurodevelopment-related genes, thereby altering microbiota composition and intestinal permeability (58, 59). Maternal immune activation (MIA), which is triggered by infections during pregnancy, is a well-established environmental risk factor for ASD (60). MIA can lead to elevated levels of pro-inflammatory cytokines in the fetal environment, which may disrupt the development of both the central nervous system and the enteric nervous system (ENS). The ENS, often termed the “second brain” regulates gut motility and secretion, and its abnormalities are frequently observed in individuals with ASD, correlating with the severity of core behavioral deficits (4, 61). Environmental factors extend beyond prenatal influences, including perinatal and postnatal exposures, and early childhood antibiotic use within the first two years of life is linked to a higher subsequent risk of both ASD and attention-deficit/hyperactivity disorder, likely through its disruptive effects on the developing gut microbiota (62, 63).

## Therapeutic implications

Microbiota-targeted interventions show promise for ameliorating both GI and behavioral symptoms. Probiotic supplementation, particularly with Bifidobacterium and Lactobacillus species, improves GI symptoms and may reduce irritability and repetitive behaviors (8, 64). A meta-analysis by Lu et al. found that gut microbiota-modulating interventions significantly reduced GI symptoms and increased the abundance of Bifidobacterium in children with ASD (65). This is supported by an updated meta-analysis of eight randomized controlled trials that demonstrated a statistically significant improvement in behavioral symptoms in the probiotic group compared with the control group (66). Subgroup analyses revealed that longer intervention durations (>3 months) and the use of multi-strain probiotics were associated with greater efficacy, underscoring the need for personalized and optimized treatment protocols (66). Microbiota transplant therapy has demonstrated sustained improvements in both GI and ASD symptoms for up to two years post-treatment (67). A recent systematic review of FMT in children and adolescents with ASD, encompassing two RCTs and seven before-and-after studies, found that while all uncontrolled studies reported symptom improvement, the results from more rigorous RCTs were inconsistent (68). One RCT showed significant improvements in GI and some behavioral symptoms compared with placebo (69), while another larger trial found no difference in core ASD symptoms between the FMT and placebo groups (70). Notably, the intervention was generally safe, with only mildly transient adverse events reported (68).

In parallel with these microbiome-based therapies, various nutritional approaches are being explored; however, elimination diets, particularly gluten-free and casein-free diets, are commonly used, although evidence for their efficacy remains inconclusive (71). While some studies reported behavioral improvements, many were limited by small sample sizes, short durations, and lack of blinding, with recent double-blind crossover trials finding no significant benefits over a regular diet (71, 72). Similarly, although a ketogenic diet may offer benefits to some, it is associated with gastrointestinal side effects and nutritional deficiencies, warranting caution and medical supervision (73). Given the high prevalence of food selectivity in ASD, which can lead to significant nutrient deficiencies, ensuring adequate intake of vitamins, such as A, D, B12, minerals, such as iron, zinc, and omega-3 fatty acids through a varied diet or supplementation is a crucial aspect of clinical management (74, 75).

## Discussion

**Figure 1:**
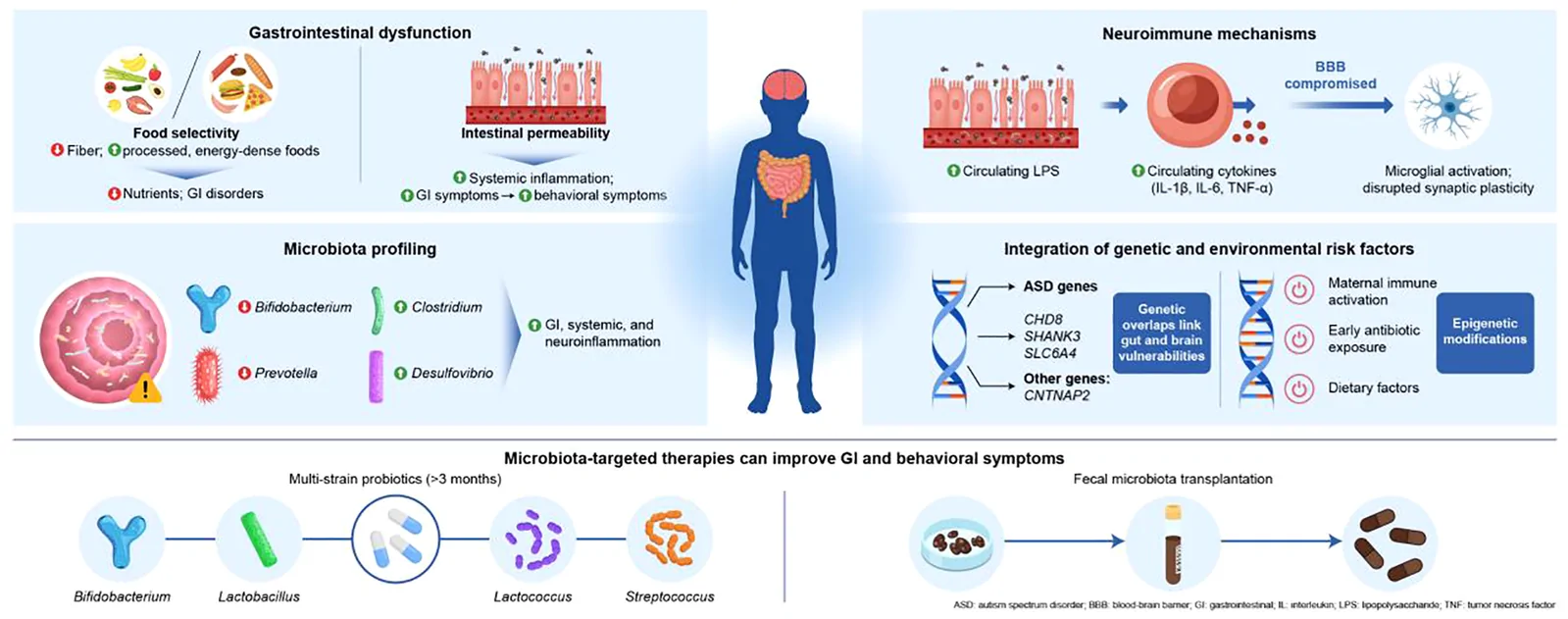
The gut-brain axis in autism spectrum disorder (ASD). This summarizes the key mechanisms linking gastrointestinal (GI) dysfunction to neurobehavioral symptoms in ASD. Food selectivity, characterized by low fiber intake and high consumption of processed, energy-dense foods, contributes to nutrient deficiencies and GI disorders. Increased intestinal permeability (“leaky gut”) facilitates the translocation of bacterial products such as lipopolysaccharide (LPS) into systemic circulation, triggering peripheral inflammation and subsequent neuroimmune activation, which may exacerbate both GI and behavioral symptoms. Altered gut microbiota in ASD includes reduced levels of Bifidobacterium and Prevotella, as well as overgrowth of *Clostridium* and *Desulfovibrio*. Microbiota-targeted therapies, including multi-strain probiotics (duration >3 months) and fecal microbiota transplantation (FMT), have shown potential in improving both GI and behavioral outcomes. ASD, autism spectrum disorder; BBB, blood-brain barrier; GI, gastrointestinal; IL, interleukin; LPS, lipopolysaccharide; TNF, tumor necrosis factor.

The gut-brain axis represents a critical interface in the pathophysiology of autism spectrum disorder, linking gastrointestinal dysfunction with core and associated behavioral symptoms. GI dysfunction in ASD is not merely a comorbidity but an integral component of the disorder, involving complex interactions among dietary patterns, microbial dysbiosis, immune activation, and genetic susceptibility (76). Unlike previous reviews that have largely focused on isolated aspects of the gut-brain axis, our synthesis integrates these components into a unified framework that emphasizes the bidirectional and self-reinforcing nature of gut-brain interactions in ASD. The convergence of genetic risk factors in the brain and gut tissues, together with environmental influences that shape microbiota composition, underscores the pleiotropic nature of ASD pathogenesis. Emerging therapeutic strategies targeting the gut microbiota, including probiotics and fecal microbiota transplantation, offer promising avenues for ameliorating both GI and behavioral symptoms, although rigorous randomized controlled trials with longer follow-up periods are needed to establish efficacy and optimize treatment protocols (77). Recent RCTs of FMT have yielded inconsistent results, highlighting the need for cautious interpretation of emerging therapeutic data (68–70). Nutritional interventions must be carefully individualized, particularly given the high prevalence of food selectivity and potential nutrient deficiencies in this population. Future research should prioritize longitudinal studies to establish causal relationships between gut dysbiosis, neuroimmune activation, and symptom progression, as well as investigations into biomarkers that could identify subgroups of individuals most likely to benefit from microbiome-targeted interventions (**Figure 1**).
